# Prognosis and Risk Factors Influencing Recurrence in Surgery-treated Patients with Primary Sacral Tumors

**Published:** 2017-08

**Authors:** Xiliang DANG, Liping LIAN, Dongsheng WU

**Affiliations:** Dept. of Orthopedics, Weinan Central Hospital, Weinan City, Shaanxi Province, China

**Keywords:** Surgical resection, Sacral tumor, Neurological function, Recurrence, Disease-free survival time

## Abstract

**Background:**

We aimed to explore the prognosis and risk factors influencing tumor recurrence in surgery-treated patients with primary sacral tumors.

**Methods:**

Fifty-six patients between February 2011 and December 2016 in Yishui Central Hospital with primary sacral tumors were selected and treated with radical surgeries. The perioperative outcomes and postoperative neurological functions were observed. After postoperative follow-up, the overall survival time (OS), disease-free survival time (DFS), and recurrence were recorded to analyze the potential risk factors influencing tumor recurrence.

**Results:**

The average surgical duration and intraoperative hemorrhagic volume were 3.92 ± 1.46 h and 2, 348.21 ± 813.67 ml, respectively. The postoperative short-term complications included three patients with infection from obstructed drainage and two with skin flap necrosis-induced infection, who recovered after anti-infection therapies; nine with incision-edge necrosis; two with calf muscle venous thrombosis; and one with an endorhachis cerebrospinal fluid fistula, who recovered after conventional treatment. Among patients, the 1-, 2- and 3-year survival rates were 91.07% (51/56), 82.14% (46/56), and 75.00% (42/56) while the 1-, 2- and 3-year DFS rates were 89.29% (50/56), 78.57% (44/56) and 71.43% (40/56), respectively. Of the 56 patients, 16 had recurrence after surgery, with recurrence rate of 28.57%. It was predicated that surgical methods and local infiltration were the independent risk factors influencing tumor recurrence (*P*<0.01).

**Conclusion:**

The reservation of bilateral S_3_ or > unilateral S_3_ nerves can improve quality of life of patients. Surgical methods and local infiltration are the independent risk factors influencing tumor recurrence, and extensive resection can effectively control the recurrence rate.

## Introduction

Primary sacral tumors are characterized by strong local infiltration, slow growth, and low rate of distant metastasis. They mainly include neurilemmoma, giant cell tumors, chondrosarcoma, and chordoma. Chordoma and giant cell tumors are the most common forms seen in clinic, accounting for 40.6% and 23.5% of all sacral tumors, respectively (1). Primary sacral tumors cause relatively low morbidity, but are insensitive to chemotherapy because of tumor heterogeneity. Therefore, surgery is the optimal therapeutic strategy (2–4). Surgery can allow for excision of tumor nidi, alleviation of pain, and prolongation of survival time, thus providing the preconditions for complementary therapies. However, because sacral tumors often invade the sacral nerves, tumor resection may result in various sequelae (including limitations of mobility and sphincter dysfunctions) and affect the quality of life (QOL) of patients. Moreover, because of the complex local anatomy and rich vasculature in the sacral region, tumors cannot be completely resected. Therefore, the recurrence rate is especially high (5, 6).

When the nerve roots above S_3_ are reserved, the secondary debridement-type extensive resection followed by stable reconstruction of lumbar vertebra and the pelvis can achieve favorable nervous and limb functions and reduce the tumor recurrence rates in patients with sacral tumors after resection (7).

To further explore the prognosis and risk factors influencing the recurrence in patients with primary sacral tumors, we observed tumor regression and neurological and limb functions, and analyzed the relationships between surgical methods, local infiltration, tumor size, and tumor patterns, with postoperative recurrence, aiming to provide a reference for the surgical treatment of patients with primary sacral tumors.

## Materials and Methods

### Patients

This study was approved by the Ethics Committee of Yishui Central Hospital and all informed consent forms were signed by the patients and their families.

A total of 56 patients pathologically diagnosed with primary sacral tumors admitted to the Department of Orthopedics of Yishui Central Hospital from February 2011 to December 2016 were selected. There were 24 males and 32 females, aged from 19–75 yr old, with average age of 45.09 ± 11.38 yr; there were 37 patients with chordoma, 13 with giant cell tumors, three with osteosarcoma, one with neurilemmoma, one with neurofibroma, and one with Ewing’s sarcoma; regarding tumor location in sacral segments, there were 38 at S_1_–S_2_ and 18 at S_3_ or below; regarding tumor size, 30 were ≤ 80 mm and 26 were >80 mm; 24 had local infiltration; nine were accompanied with different degrees of prolapse of the lumbar intervertebral disc; the period from the initial onset of symptoms until diagnosis was 2–13 months, with average of 6.53 ± 3.14 months; and preoperative symptoms included 45 with lumbosacral pain, 20 with radiating pain in the lower legs, five with sensory and motor dysfunction in the lower limbs, and two with fecal and urinary disturbance.

### Methods

Surgical methods: Gastrointestinal disinfectant was used 2 d before surgery to conduct the cleansing enema. General anesthesia or continuous epidural anesthesia was performed. The surgical approaches were chosen according to tumor size and location as follows: 1. The anterior approach was performed in patients with tumors above the S_2_ level or growing toward the pelvis, and tumors in the pelvis were resected through an extraperitoneal approach; 2. The posterior approach was conducted in patients with tumors at or below the S_3_ level. The patients were placed in the prone position. Large arc-shaped or I-shaped incisions were made to expose the back wall of the tumor. The tailbone was excised. Blunt dissection of the anterior sacrum was performed. Gauze was used. We dissociated the tumor and the front tissues, and the tumors and sacral bones were then resected; 3. The anterior-posterior approach was applied in patients with a large anterior pump involving S_1_–S_2_. Patients were placed in the supine position. The internal iliac artery and median sacral artery were ligated outside the peritoneal cavity through the lower abdomen. Pelvic organs and vessels were isolated, and the front-walls of tumors were dissociated. Next, the anterior approach was applied to resect tumors after patients were placed in the lateral decubitus position. Extensive resection was adopted in patients with the upper tumor edge at the S_3_ level or below. For patients with the upper tumor edge above the S_3_ level, or edges at the S_3_ level and above were resected, and the proximal part of tumors was scraped. For patients with higher levels of sacral bone that required resection, if S_1_ vertebra were to be excised, pelvic stability was reconstructed. During surgery, bilateral S_3_ or > unilateral S_3_ nerves were reserved as much as possible.

Follow-up: Fecal and urinary functions and sensory and motor functions of the lower limbs were followed-up every 6 months after surgery. Imageological examinations such as X-ray, computed tomography, or magnetic resonance imaging were re-examined once every 3 months for the first year, once every 6 months for the second year, and then once per year. The follow-up time ended in December 2015 or until the death of the patient.

### Observational indexes

The intraoperative outcomes, such as surgical methods, intraoperative hemorrhagic volume, surgical duration, and postoperative complications were observed. Postoperative neurological functions including fecal and urinary functions, and the sensory and motor functions of the lower limbs were observed. The overall survival (OS), disease-free survival (DFS), and recurrence rates were recorded.

### Data analysis

SAS9.3 and SPSS17.0 software (Chicago, IL, USA) were used for data analysis. Measurement data are presented as mean ± standard deviation, independent-sample *t-*test was used for paired comparisons while variance analysis was used for multi-sample comparisons. For classified variables, the frequency and percentage of each pattern were calculated, and detected by a χ^2^ test or continuous calibration χ^2^ test, while classification variables were detected with a χ^2^ trend test. The life table method was used for survival analysis. Cox stepwise regression analysis was used to determine the independent risk factors influencing the recurrence rate of sacral tumors. All tests were bilateral and *P*<0.05 was considered statistically significant.

## Results

### Perioperative outcomes

All patients completed surgery and none died during surgery. The surgical duration was 2–7.5 h, with average of 3.92 ± 1.46 h and the intra-operative hemorrhagic volume was 200–5700 ml, with average of 2348.21 ± 813.67 ml. Comparison of interoperative outcomes with the different surgical methods showed that surgical duration was significantly longer with the anterior-posterior approach compared with the other two approaches (*P*<0.01). The intraoperative hemorrhagic volume was significantly higher with the anterior-posterior approach compared with the anterior approach (*P*<0.05), although there were no significant differences between the anterior approach and single posterior approach in surgical duration and intraoperative hemorrhagic volume (*P*>0.05) (Table 1).

**Table 1:** Comparison of intraoperative outcomes with different surgical methods (x±s)

**Variable**	**n**	**Surgical duration (min)**	**Intraoperative hemorrhagic volume (ml)**
Anterior approach	13	142.31±63.17	1984.62±735.96
Single posterior approach	16	179.38±71.09	2162.50±801.42
Anterior-posterior approach	27	312.96±114.35^**##^	2633.33±829.49^*^
*F* value		18.5910	3.4779
*P* value		0.0000	0.0381

Compared with anterior approach, *P<0.05, **P<0.01;

Compared with single posterior approach, ##*P<*0.01.

The postoperative short-term complications included three patients with infection from obstructed drainage and two with skin flap necrosis-induced infection, who recovered after anti-infection therapies; nine with incision-edge necrosis; two with calf muscle venous thrombosis; and one with an endorhachis cerebrospinal fluid fistula, who recovered after conventional treatment.

### Postoperative neurological function

Thirty-three patients, whose bilateral S_4_–S_5_ nerve roots were resected, had completely recovered fecal and urinary functions. Among them, 27 (81.82%) patients had completely recovered bladder function; Of the 17 patients with unilateral S_3_ and bilateral S_4—5_ nerve roots resected, 11 (64.71%) had completely recovered fecal and urinary functions within the 6-month postoperative period. Of the six patients with bilateral nerve roots resected, two (33.33%) had recovered fecal and urinary functions and two (33.33%) had recovered bladder function after surgery. No patients had sensory and motor dysfunction of the lower limbs.

### Survival analysis

**Fig. 1:.**
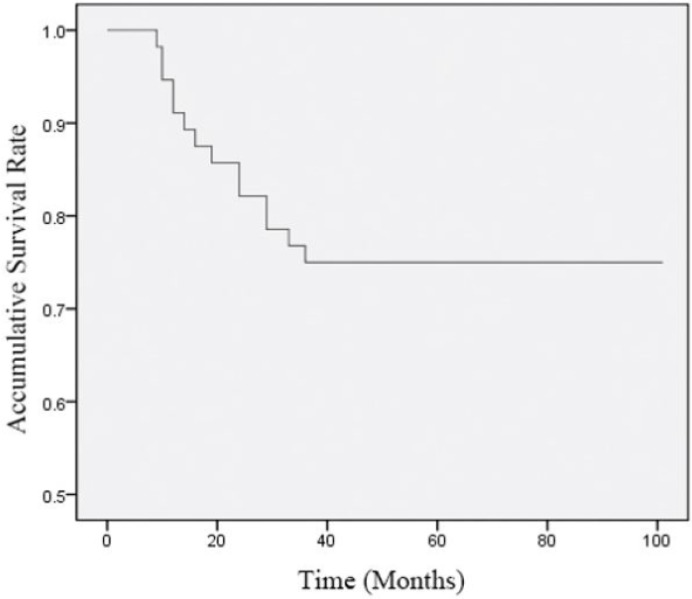
Curve of Accumulative Survival Rate. The follow-up period was 8–106 months, and the survival rate decreased as the follow-up time increased. For all patients, the 1-, 2- and 3-year survival rates were 91.07% (51/56), 82.14% (46/56), and 75.00% (42/56) respectively

**Fig. 2:.**
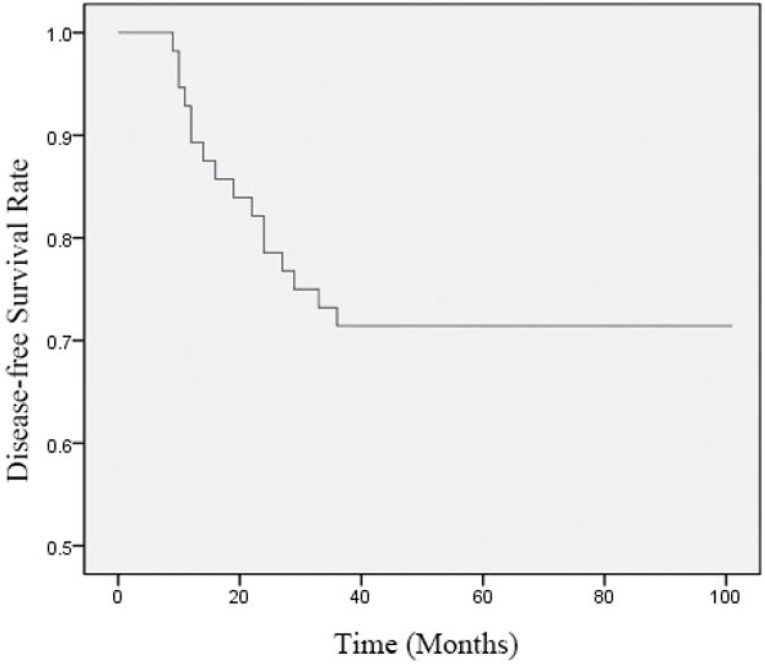
Curve of Disease-free Survival (DFS) Rate. The follow-up period was 8–106 months, with average time and follow-up rate of 83.02 ± 22.85 months and 100%, respectively. The DFS rate decreased as the follow-up time increased. For all patients, the 1-, 2- and 3-year DFS rates were 89.29% (50/56), 78.57% (44/56), and 71.43% (40/56), respectively

The follow-up period of this study was 8–106 months, with average time and follow-up rate of 83.02 ± 22.85 months and 100%, respectively. In all patients, the 1-, 2- and 3-year survival rates were 91.07% (51/56), 82.14% (46/56), and 75.00% (42/56), while the 1-, 2- and 3-year DFS rates were 89.29% (50/56), 78.57% (44/56), and 71.43% (40/56), respectively (Fig. 1 and 2).

### Analysis of risk factors influencing tumor recurrence

Among all patients, 16 had postoperative recurrence, with recurrence rate of 28.57%. The univariate analysis demonstrated that tumor location, the presence of local infiltration, and surgical methods were in close correlation with postoperative recurrence (*P*<0.05 or *P*<0.01), as shown in Table 2. The multivariate analysis was further evaluated by the Cox regression model, the results of which indicated that surgical methods and the presence of local infiltration were the independent risk factors influencing the prognosis (*P*<0.01) (Table 3).

**Table 2:** Univariate analysis of postoperative recurrence of sacral tumors

	**n**	**Recurrence [n(%)]**	**Non-recurrence [n(%)]**	***χ*^2^ value**	***P* value**
Tumor patterns					
Chordoma	37	13(35.14)	24(64.86)	2.3054	0.3158
Giant cell tumor	13	2(15.38)	11(84.62)		
Others	6	1(16.67)	5(83.33)		
Tumor location involved sacral segments					
S_1_∼S_2_	38	14(36.84)	24(63.16)	3.9626	0.0465
S_3_ or below	18	2(11.11)	16(88.89)		
Tumor size (mm)					
≤80	30	8(26.67)	22(73.33)	0.1149	0.7347
>80	26	8(30.77)	18(69.23)		
Local infiltration					
Yes	24	13(54.17)	11(45.83)	13.4823	0.0002
No	32	3(9.38)	29(90.63)		
Surgical methods (1)					
Anterior approach	13	5(38.46)	8(61.54)	2.6801	0.2618
Single posterior approach	16	2(12.50)	14(87.50)		
Anterior-posterior approach	27	8(29.63)	19(70.37)		
Surgical methods (2)					
Scrape	11	7(63.64)	4(36.36)	10.5473	0.0051
Extensive resection	16	1(6.25)	15(93.75)		
Resection+scrape	29	8(27.59)	21(72.41)		

**Table 3:** Multivariate analysis of postoperative recurrence of sacral tumors

**Influencing factors**	**Regression coefficient**	***P* value**	**Relative risk (RR)**	**95% confidence interval**	
				Lower limit	Upper limit
Local infiltration	1.799	0.009	7.826	1.604	36.988
Surgical methods (2)	2.101	0.006	6.083	1.619	22.815

## Discussion

Primary sacral tumors located deep inside the human body grow slowly and remain dormant until obvious manifestation occur. Damage of the sacral bone is often observed when patients are diagnosed. Moreover, because most sacral tumors grow toward the pelvis, they can easily invade the cauda equine, thus involving the sacral nerve roots (8). Surgical treatment is the optimal therapeutic strategy for most patients with primary sacral tumors, especially those with sacral tumors in low locations, because when tumors are benign or have low have degree of malignancy, the clinical efficacy of surgery will be better. However, during the treatment of patients with sacral tumors in a high location, the reservation of sacral nerve roots is involved.

Sacral tumors are difficult to be completely exposed during the surgical process, so they cannot be radically resected. Additionally, even if they can be completely resected, they can still cause various degrees of damage to abdominal organs (i.e. colorectum) or sacrifice most of the sacral nerves, which would greatly reduce the postoperative QOL of patients (9, 10).

The postoperative effect of surgery on patients with primary sacral tumors can be influenced by two main aspects: the absence of sacral bones, given their importance on stability of the pelvis and spin, which can recover after internal-fixation reconstruction (11); in addition, there is postoperative dysfunction and mobility limitations of sphincters, which are associated with the preservation of sacral nerve roots during surgery. A previous study found that conventional therapy could effectively reduce dysfunction of the bladder, intestine, and limbs, and the postoperative QOL of patients was closely correlated with the number of preserved nerve roots (12, 13). In the present study, ≥ unilateral S_3_ nerves were preserved and the bladder function of most patients recovered within 6 months. Therefore, the principle for reserving nerve roots was that bilateral S_1_–_2_ or ≥ unilateral S_3_ nerve roots should be reserved under the condition that complete resection of tumors was not affected.

Sacral tumors occur in multiple patterns and have a high recurrence rate. The reasons for recurrence include 1) The tumor cells grow through muscular intervals toward the gluteus muscle; 2) The surgical resection does not reach the surgical edge of the tumor; 3) The tumor cells invade the surgical wounds and soft tissues during surgery; 4) Postoperative radiochemotherapy is not conducted or has poor clinical efficacy (14). Ming et al. (15) applied preoperative embolization combined with surgical resection for the treatment of patients with sacral giant cell tumors. Their results showed that the 3- and 5-year DFS rates were 89.1% and 75.5%, respectively, with recurrence rate of 28.6%, and further analysis of the risk factors influencing tumor recurrence illustrated that tumor recurrence was closely correlated with tumor stage, but had no correlation with age, gender, tumor location, tumor size, or the use of chemotherapy. Li et al. (12) performed surgical resection in 32 patients with sacral giant cell tumors, demonstrating that the recurrence rates of patients undergoing extensive resection and scrape were 18.2% and 71.4%, respectively, and the difference was significant. In the present study, the tumor recurrence rate was 28.57%, and univariate and multivariate analyses suggested that surgical methods and the presence of local infiltration were the independent risk factors influencing tumor recurrence, which were consistent with the results of previous studies.

## Conclusion

Surgical resection has favorable clinical efficacy for the treatment of sacral tumors, and reservation of bilateral S_3_ or ≥ unilateral S_3_ nerves can improve the QOL of patients. Additionally, surgical methods and the presence of local infiltration are the independent risk factors influencing tumor recurrence, and extensive resection can effectively control the tumor recurrence rate and improve the prognosis of patients with primary sacral tumors.

## Ethical considerations

Ethical issues (Including plagiarism, informed consent, misconduct, data fabrication and/or falsification, double publication and/or submission, redundancy, etc.) have been completely observed by the authors.
